# 2,4,6-Trimethyl­pyridinium dihydrogen phosphate

**DOI:** 10.1107/S1600536810052153

**Published:** 2010-12-18

**Authors:** Jing Dai, Jie Xu

**Affiliations:** a Ordered Matter Science Research Center, College of Chemistry and Chemical Engineering, Southeast University, Nanjing 210096, People’s Republic of China

## Abstract

The asymmetric unit of the title compound, C_8_H_12_N^+^·H_2_PO_4_^−^, contains two H_2_PO_4_^−^ anions and two 2,4,6-trimethyl­pyridinium cations. In the crystal, the anions are linked by O—H⋯O hydrogen bonds, forming supra­molecular chains running along the *a* axis; the cations are connected to the anion chains by N—H⋯O hydrogen bonds. Weak inter­molecular C—H⋯O hydrogen bonding is also present in the crystal structure.

## Related literature

For the properties and structures of pyridine salts, see: Fu *et al.* (2007 ▶, 2008 ▶, 2009 ▶); Fu & Xiong (2008 ▶).
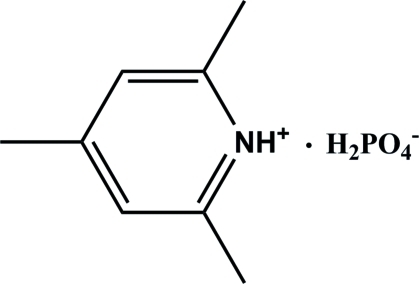


## Experimental

### 

#### Crystal data

C_8_H_12_N^+^·H_2_PO_4_^−^
*M* *_r_* = 219.17
Monoclinic, 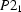

*a* = 7.9501 (16) Å
*b* = 15.324 (3) Å
*c* = 9.0252 (18) Å
β = 97.97 (3)°
*V* = 1088.9 (4) Å^3^
*Z* = 4
Mo *K*α radiation
μ = 0.24 mm^−1^
*T* = 298 K
0.30 × 0.05 × 0.05 mm

#### Data collection

Rigaku Mercury2 diffractometer
Absorption correction: multi-scan (*CrystalClear*; Rigaku, 2005 ▶) *T*_min_ = 0.910, *T*_max_ = 1.000
11306 measured reflections
4970 independent reflections
3973 reflections with *I* > 2σ(*I*)
*R*_int_ = 0.033

#### Refinement

*R*[*F* ^2^ > 2σ(*F* ^2^)] = 0.044
*wR*(*F* ^2^) = 0.101
*S* = 1.02
4970 reflections
275 parameters
5 restraints
H atoms treated by a mixture of independent and constrained refinement
Δρ_max_ = 0.15 e Å^−3^
Δρ_min_ = −0.25 e Å^−3^
Absolute structure: Flack (1983 ▶), 2380 Friedel pairs
Flack parameter: 0.01 (8)

### 

Data collection: *CrystalClear* (Rigaku, 2005 ▶); cell refinement: *CrystalClear*; data reduction: *CrystalClear*; program(s) used to solve structure: *SHELXTL* (Sheldrick, 2008 ▶); program(s) used to refine structure: *SHELXTL*; molecular graphics: *SHELXTL*; software used to prepare material for publication: *SHELXTL*.

## Supplementary Material

Crystal structure: contains datablocks I, global. DOI: 10.1107/S1600536810052153/xu5121sup1.cif

Structure factors: contains datablocks I. DOI: 10.1107/S1600536810052153/xu5121Isup2.hkl

Additional supplementary materials:  crystallographic information; 3D view; checkCIF report

## Figures and Tables

**Table 1:** Hydrogen-bond geometry (Å, °)

*D*—H⋯*A*	*D*—H	H⋯*A*	*D*⋯*A*	*D*—H⋯*A*
O1—H1⋯O8	0.85 (2)	1.77 (2)	2.610 (2)	169 (3)
O3—H3⋯O7^i^	0.85 (3)	1.72 (3)	2.569 (2)	173 (3)
O5—H5⋯O4^ii^	0.84 (2)	1.79 (2)	2.627 (3)	173 (3)
O6—H6⋯O2	0.85 (2)	1.69 (2)	2.538 (2)	176 (3)
N1—H1*A*⋯O4^iii^	0.86	1.77	2.633 (3)	177
N2—H2*A*⋯O8^iv^	0.86	1.78	2.632 (3)	170
C2—H2*B*⋯O6^v^	0.93	2.59	3.443 (4)	152
C4—H4*A*⋯O7^i^	0.93	2.59	3.481 (4)	161

Symmetry codes: (i) 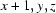; (ii) 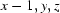; (iii) 
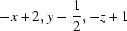
; (iv) 
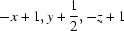
; (v) 
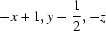
.

**Fig. 1.**
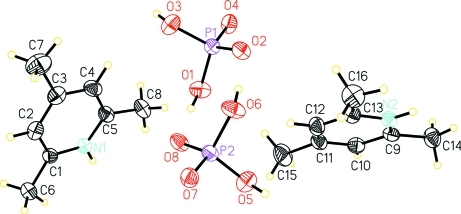
A view of the title compound with the atomic numbering scheme. Displacement ellipsoids were drawn at the 30% probability level.

**Fig. 2.**
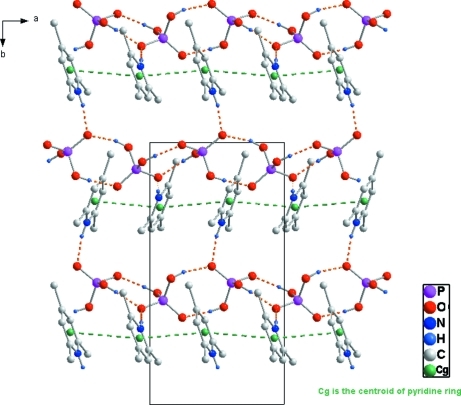
The crystal packing of the title compound, showing the two-dimensional network. H atoms not involved in hydrogen bonding (dashed line) have been omitted for clarity.

## supplementary crystallographic information

### Comment

Salts of pyridine attracted more attention as phase transition dielectric materials for its application in memory storage (Fu *et al.* 2007; Fu & Xiong 2008; Fu *et al.* 2008; Fu *et al.* 2009). With the purpose of obtaining phase transition crystals of 2,4,6-trimethylpyridine salts, its interaction with various acids has been studied and we have elaborated a series of new materials with this organic molecule. In this study, we describe the crystal structure of the title compound, di-2,4,6-trimethylpyridinium dihydrogen phosphate.

The dielectric constant of title compound as a function of temperature indicates that the permittivity is basically temperature-independent, suggesting that this compound should be not a real ferroelectrics or there may be no distinct phase transition occurred within the measured temperature range. Similarly, below the melting point (425 K) of the compound, the dielectric constant as a function of temperature also goes smoothly, and there is no dielectric anomaly observed (dielectric constant equaling to 6.9 to 8.7).

The asymmetric unit is composed of two H_2_PO_4_^-^ anion and two C_8_H_12_N^+^ cation (Fig.1). Both the pyridine N atoms are protonated, thus indicating two positive charges in the pyridine N atoms. And the H_2_PO_4_^-^ anions were showing two negative charges to make the charge balance. The geometric parameters of the title compound are in the normal range.

In the crystal structure, all the H atoms of pyridinium cations and the H_2_PO_4_^-^ anions are involved in N—H···O and O—H···O hydrogen bonds. These hydrogen bonds link the ionic units into a one-dimentional chains parallel to the *a*-axis. Furthermore, the π···π (centroid-to-centroid distance = 3.8403 (8) Å and 4.1676 (8) Å) interactions link the chains into a two-dimentional network parallel to the (0 0 1) plane. (Table 1 and Fig.2).

### Experimental

The commercial 2,4,6-trimethylpyridine (3 mmol) was dissolved in water/H_3_PO_4_ (50:1 *v*/*v*) solution. The solvent was slowly evaporated in air affording colourless needle-shaped crystals of the title compound suitable for X-ray analysis.

### Refinement

H atoms of H_2_PO _4_^-^ anions were located in a difference Fourier map and freely refined, with the O—H distance constrained to 0.85 Å. Other H atoms were fixed geometrically and treated as riding with C–H = 0.93 Å (aromatic), 0.96 Å (methyl) and N–H = 0.86 Å, with *U*_iso_(H) = 1.2U_eq_(C,N) and *U*_iso_(H) = 1.5U_eq_(C) for methyl.

### Crystal data

**Table:**

C_8_H_12_N^+^·H_2_PO_4_^−^	*F*(000) = 464
*M**_r_* = 219.17	*D*_x_ = 1.337 Mg m^−^^3^
Monoclinic, *P*2_1_	Mo *K*α radiation, λ = 0.71073 Å
Hall symbol: P 2yb	Cell parameters from 4970 reflections
*a* = 7.9501 (16) Å	θ = 3.2–27.5°
*b* = 15.324 (3) Å	µ = 0.24 mm^−^^1^
*c* = 9.0252 (18) Å	*T* = 298 K
β = 97.97 (3)°	Needle, colorless
*V* = 1088.9 (4) Å^3^	0.30 × 0.05 × 0.05 mm
*Z* = 4	

### Data collection

**Table:**

Rigaku Mercury2 diffractometer	4970 independent reflections
Radiation source: fine-focus sealed tube	3973 reflections with *I* > 2σ(*I*)
graphite	*R*_int_ = 0.033
Detector resolution: 13.6612 pixels mm^-1^	θ_max_ = 27.5°, θ_min_ = 3.2°
CCD profile fitting scans	*h* = −10→10
Absorption correction: multi-scan (*CrystalClear*; Rigaku, 2005)	*k* = −19→19
*T*_min_ = 0.910, *T*_max_ = 1.000	*l* = −11→11
11306 measured reflections	

### Refinement

**Table:**

Refinement on *F*^2^	Secondary atom site location: difference Fourier map
Least-squares matrix: full	Hydrogen site location: inferred from neighbouring sites
*R*[*F*^2^ > 2σ(*F*^2^)] = 0.044	H atoms treated by a mixture of independent and constrained refinement
*wR*(*F*^2^) = 0.101	*w* = 1/[σ^2^(*F*_o_^2^) + (0.0489*P*)^2^ + 0.0746*P*] where *P* = (*F*_o_^2^ + 2*F*_c_^2^)/3
*S* = 1.02	(Δ/σ)_max_ = 0.001
4970 reflections	Δρ_max_ = 0.15 e Å^−^^3^
275 parameters	Δρ_min_ = −0.25 e Å^−^^3^
5 restraints	Absolute structure: Flack (1983), 2380 Friedel pairs
Primary atom site location: structure-invariant direct methods	Flack parameter: 0.01 (8)

### Special details

**Table:**

Geometry. All e.s.d.'s (except the e.s.d. in the dihedral angle between two l.s. planes) are estimated using the full covariance matrix. The cell e.s.d.'s are taken into account individually in the estimation of e.s.d.'s in distances, angles and torsion angles; correlations between e.s.d.'s in cell parameters are only used when they are defined by crystal symmetry. An approximate (isotropic) treatment of cell e.s.d.'s is used for estimating e.s.d.'s involving l.s. planes.
Refinement. Refinement of *F*^2^ against ALL reflections. The weighted *R*-factor *wR* and goodness of fit *S* are based on *F*^2^, conventional *R*-factors *R* are based on *F*, with *F* set to zero for negative *F*^2^. The threshold expression of *F*^2^ > σ(*F*^2^) is used only for calculating *R*-factors(gt) *etc*. and is not relevant to the choice of reflections for refinement. *R*-factors based on *F*^2^ are statistically about twice as large as those based on *F*, and *R*- factors based on ALL data will be even larger.

### Fractional atomic coordinates and isotropic or equivalent isotropic displacement parameters (Å^2^)

**Table:**

	*x*	*y*	*z*	*U*_iso_*/*U*_eq_	
P1	0.90061 (8)	0.60246 (4)	0.30479 (7)	0.04278 (17)	
P2	0.39135 (8)	0.53464 (4)	0.24480 (8)	0.04306 (17)	
O1	0.8423 (2)	0.51862 (14)	0.3826 (2)	0.0600 (5)	
N1	0.9315 (3)	0.21030 (14)	0.3374 (2)	0.0439 (5)	
H1A	0.9367	0.1865	0.4242	0.053*	
O5	0.3427 (2)	0.54241 (16)	0.4065 (2)	0.0618 (5)	
O2	0.7595 (2)	0.66752 (12)	0.2811 (2)	0.0532 (5)	
N2	0.5339 (3)	0.80459 (14)	0.7547 (2)	0.0445 (5)	
H2A	0.4972	0.8574	0.7515	0.053*	
O6	0.4507 (2)	0.62723 (13)	0.2008 (3)	0.0632 (6)	
O3	0.9432 (2)	0.57407 (16)	0.1485 (2)	0.0602 (6)	
O7	0.2392 (2)	0.50926 (13)	0.1375 (2)	0.0558 (5)	
O4	1.0612 (2)	0.63287 (13)	0.4013 (2)	0.0561 (5)	
O8	0.5398 (2)	0.47211 (12)	0.2575 (2)	0.0561 (5)	
C8	1.0619 (4)	0.3360 (2)	0.4720 (3)	0.0654 (8)	
H8A	1.1303	0.2957	0.5356	0.098*	
H8B	0.9697	0.3560	0.5215	0.098*	
H8C	1.1301	0.3849	0.4506	0.098*	
C13	0.5302 (3)	0.76072 (19)	0.6246 (3)	0.0481 (6)	
C5	0.9927 (3)	0.29160 (17)	0.3293 (3)	0.0490 (6)	
C1	0.8620 (3)	0.16344 (18)	0.2166 (3)	0.0459 (6)	
C12	0.5901 (4)	0.6770 (2)	0.6307 (3)	0.0565 (7)	
H12A	0.5901	0.6457	0.5424	0.068*	
C9	0.5917 (3)	0.77016 (17)	0.8891 (3)	0.0456 (6)	
C10	0.6513 (3)	0.68578 (18)	0.8946 (3)	0.0512 (7)	
H10A	0.6924	0.6609	0.9865	0.061*	
C2	0.8552 (4)	0.2015 (2)	0.0782 (3)	0.0553 (7)	
H2B	0.8070	0.1712	−0.0064	0.066*	
C6	0.8017 (4)	0.0734 (2)	0.2423 (3)	0.0633 (8)	
H6A	0.8924	0.0404	0.2973	0.095*	
H6B	0.7662	0.0456	0.1478	0.095*	
H6C	0.7078	0.0760	0.2985	0.095*	
C11	0.6509 (3)	0.63792 (17)	0.7658 (3)	0.0526 (7)	
C14	0.5867 (5)	0.8266 (2)	1.0239 (3)	0.0691 (9)	
H14A	0.6357	0.8824	1.0076	0.104*	
H14B	0.6501	0.7993	1.1097	0.104*	
H14C	0.4710	0.8342	1.0407	0.104*	
C3	0.9196 (4)	0.2845 (2)	0.0636 (3)	0.0580 (7)	
C4	0.9881 (4)	0.3288 (2)	0.1899 (3)	0.0585 (7)	
H4A	1.0319	0.3846	0.1812	0.070*	
C15	0.7111 (5)	0.5453 (2)	0.7726 (4)	0.0814 (10)	
H15A	0.7847	0.5357	0.8648	0.122*	
H15B	0.7721	0.5339	0.6900	0.122*	
H15C	0.6152	0.5068	0.7673	0.122*	
C16	0.4597 (4)	0.8072 (2)	0.4839 (3)	0.0729 (9)	
H16A	0.4441	0.8678	0.5054	0.109*	
H16B	0.3524	0.7819	0.4443	0.109*	
H16C	0.5372	0.8018	0.4119	0.109*	
C7	0.9177 (5)	0.3237 (3)	−0.0919 (4)	0.0923 (12)	
H7A	0.9096	0.3861	−0.0860	0.138*	
H7B	0.8218	0.3015	−0.1575	0.138*	
H7C	1.0204	0.3082	−0.1302	0.138*	
H5	0.254 (2)	0.5713 (15)	0.413 (3)	0.048 (8)*	
H1	0.745 (2)	0.4985 (19)	0.350 (3)	0.060 (9)*	
H6	0.5523 (18)	0.643 (2)	0.229 (3)	0.072 (11)*	
H3	1.037 (3)	0.549 (2)	0.142 (4)	0.089 (13)*	

### Atomic displacement parameters (Å^2^)

**Table:**

	*U*^11^	*U*^22^	*U*^33^	*U*^12^	*U*^13^	*U*^23^
P1	0.0381 (3)	0.0457 (4)	0.0429 (4)	−0.0005 (3)	0.0001 (3)	0.0003 (3)
P2	0.0388 (3)	0.0379 (3)	0.0519 (4)	−0.0018 (3)	0.0045 (3)	−0.0010 (3)
O1	0.0492 (11)	0.0587 (14)	0.0693 (13)	0.0007 (11)	−0.0019 (10)	0.0212 (10)
N1	0.0441 (11)	0.0414 (12)	0.0453 (12)	0.0021 (10)	0.0025 (9)	0.0010 (9)
O5	0.0510 (11)	0.0827 (15)	0.0503 (11)	0.0086 (12)	0.0020 (9)	−0.0016 (11)
O2	0.0446 (11)	0.0436 (10)	0.0695 (13)	0.0002 (9)	0.0014 (9)	0.0022 (9)
N2	0.0467 (12)	0.0372 (11)	0.0492 (13)	0.0005 (10)	0.0053 (10)	0.0018 (9)
O6	0.0416 (11)	0.0469 (12)	0.0982 (16)	−0.0006 (9)	−0.0008 (11)	0.0205 (10)
O3	0.0453 (11)	0.0892 (16)	0.0445 (11)	0.0043 (11)	−0.0002 (8)	−0.0064 (10)
O7	0.0446 (10)	0.0650 (13)	0.0565 (11)	−0.0038 (9)	0.0023 (8)	−0.0165 (9)
O4	0.0421 (10)	0.0681 (13)	0.0556 (11)	0.0002 (9)	−0.0022 (8)	−0.0160 (9)
O8	0.0424 (10)	0.0383 (10)	0.0862 (14)	0.0005 (9)	0.0034 (9)	−0.0012 (9)
C8	0.074 (2)	0.0488 (16)	0.0685 (19)	−0.0032 (16)	−0.0079 (16)	−0.0075 (15)
C13	0.0465 (15)	0.0522 (16)	0.0439 (15)	−0.0034 (13)	0.0000 (11)	−0.0035 (12)
C5	0.0447 (14)	0.0375 (14)	0.0641 (18)	0.0052 (12)	0.0048 (12)	0.0029 (12)
C1	0.0396 (13)	0.0469 (15)	0.0506 (15)	−0.0012 (12)	0.0039 (11)	−0.0048 (12)
C12	0.0620 (17)	0.0548 (17)	0.0520 (16)	−0.0010 (15)	0.0051 (13)	−0.0139 (14)
C9	0.0485 (15)	0.0424 (14)	0.0462 (15)	−0.0074 (12)	0.0072 (11)	0.0034 (11)
C10	0.0540 (16)	0.0512 (16)	0.0476 (15)	−0.0020 (13)	0.0044 (12)	0.0129 (13)
C2	0.0521 (15)	0.0615 (19)	0.0521 (17)	0.0062 (15)	0.0065 (12)	−0.0039 (14)
C6	0.0656 (19)	0.0533 (18)	0.072 (2)	−0.0094 (15)	0.0119 (15)	−0.0097 (14)
C11	0.0472 (15)	0.0407 (15)	0.0696 (19)	0.0005 (12)	0.0074 (13)	−0.0007 (13)
C14	0.102 (3)	0.0580 (18)	0.0482 (17)	−0.0005 (19)	0.0136 (16)	−0.0051 (14)
C3	0.0587 (17)	0.0607 (19)	0.0554 (17)	0.0128 (15)	0.0103 (13)	0.0066 (14)
C4	0.0590 (17)	0.0442 (15)	0.072 (2)	0.0042 (14)	0.0098 (15)	0.0080 (15)
C15	0.087 (2)	0.0502 (19)	0.105 (3)	0.0163 (18)	0.009 (2)	0.0011 (18)
C16	0.088 (2)	0.073 (2)	0.0536 (18)	0.0057 (19)	−0.0051 (17)	0.0082 (16)
C7	0.107 (3)	0.098 (3)	0.073 (2)	0.012 (3)	0.015 (2)	0.034 (2)

### Geometric parameters (Å, °)

**Table:**

P1—O2	1.494 (2)	C12—C11	1.384 (4)
P1—O4	1.5155 (19)	C12—H12A	0.9300
P1—O3	1.558 (2)	C9—C10	1.376 (4)
P1—O1	1.565 (2)	C9—C14	1.498 (4)
P2—O7	1.4924 (19)	C10—C11	1.374 (4)
P2—O8	1.5120 (19)	C10—H10A	0.9300
P2—O6	1.564 (2)	C2—C3	1.385 (4)
P2—O5	1.565 (2)	C2—H2B	0.9300
O1—H1	0.849 (10)	C6—H6A	0.9600
N1—C5	1.343 (3)	C6—H6B	0.9600
N1—C1	1.358 (3)	C6—H6C	0.9600
N1—H1A	0.8600	C11—C15	1.497 (4)
O5—H5	0.843 (10)	C14—H14A	0.9600
N2—C9	1.343 (3)	C14—H14B	0.9600
N2—C13	1.351 (3)	C14—H14C	0.9600
N2—H2A	0.8600	C3—C4	1.373 (4)
O6—H6	0.849 (10)	C3—C7	1.525 (4)
O3—H3	0.85 (3)	C4—H4A	0.9300
C8—C5	1.492 (4)	C15—H15A	0.9600
C8—H8A	0.9600	C15—H15B	0.9600
C8—H8B	0.9600	C15—H15C	0.9600
C8—H8C	0.9600	C16—H16A	0.9600
C13—C12	1.367 (4)	C16—H16B	0.9600
C13—C16	1.495 (4)	C16—H16C	0.9600
C5—C4	1.377 (4)	C7—H7A	0.9600
C1—C2	1.372 (4)	C7—H7B	0.9600
C1—C6	1.490 (4)	C7—H7C	0.9600
			
O2—P1—O4	115.66 (12)	C11—C10—H10A	119.7
O2—P1—O3	108.04 (12)	C9—C10—H10A	119.7
O4—P1—O3	109.57 (11)	C1—C2—C3	120.6 (3)
O2—P1—O1	110.44 (11)	C1—C2—H2B	119.7
O4—P1—O1	105.82 (11)	C3—C2—H2B	119.7
O3—P1—O1	106.98 (13)	C1—C6—H6A	109.5
O7—P2—O8	115.98 (11)	C1—C6—H6B	109.5
O7—P2—O6	108.51 (11)	H6A—C6—H6B	109.5
O8—P2—O6	109.55 (10)	C1—C6—H6C	109.5
O7—P2—O5	109.99 (11)	H6A—C6—H6C	109.5
O8—P2—O5	105.56 (12)	H6B—C6—H6C	109.5
O6—P2—O5	106.86 (13)	C10—C11—C12	118.3 (3)
P1—O1—H1	117 (2)	C10—C11—C15	120.5 (3)
C5—N1—C1	123.9 (2)	C12—C11—C15	121.1 (3)
C5—N1—H1A	118.1	C9—C14—H14A	109.5
C1—N1—H1A	118.1	C9—C14—H14B	109.5
P2—O5—H5	115.0 (17)	H14A—C14—H14B	109.5
C9—N2—C13	123.6 (2)	C9—C14—H14C	109.5
C9—N2—H2A	118.2	H14A—C14—H14C	109.5
C13—N2—H2A	118.2	H14B—C14—H14C	109.5
P2—O6—H6	119 (2)	C4—C3—C2	119.0 (3)
P1—O3—H3	120 (2)	C4—C3—C7	121.4 (3)
C5—C8—H8A	109.5	C2—C3—C7	119.6 (3)
C5—C8—H8B	109.5	C3—C4—C5	120.7 (3)
H8A—C8—H8B	109.5	C3—C4—H4A	119.6
C5—C8—H8C	109.5	C5—C4—H4A	119.6
H8A—C8—H8C	109.5	C11—C15—H15A	109.5
H8B—C8—H8C	109.5	C11—C15—H15B	109.5
N2—C13—C12	117.8 (2)	H15A—C15—H15B	109.5
N2—C13—C16	117.5 (3)	C11—C15—H15C	109.5
C12—C13—C16	124.7 (3)	H15A—C15—H15C	109.5
N1—C5—C4	118.0 (3)	H15B—C15—H15C	109.5
N1—C5—C8	117.9 (3)	C13—C16—H16A	109.5
C4—C5—C8	124.0 (3)	C13—C16—H16B	109.5
N1—C1—C2	117.8 (3)	H16A—C16—H16B	109.5
N1—C1—C6	118.0 (2)	C13—C16—H16C	109.5
C2—C1—C6	124.2 (3)	H16A—C16—H16C	109.5
C13—C12—C11	121.3 (3)	H16B—C16—H16C	109.5
C13—C12—H12A	119.4	C3—C7—H7A	109.5
C11—C12—H12A	119.4	C3—C7—H7B	109.5
N2—C9—C10	118.4 (2)	H7A—C7—H7B	109.5
N2—C9—C14	117.5 (2)	C3—C7—H7C	109.5
C10—C9—C14	124.1 (2)	H7A—C7—H7C	109.5
C11—C10—C9	120.7 (3)	H7B—C7—H7C	109.5
			
C9—N2—C13—C12	−0.6 (4)	N1—C1—C2—C3	0.8 (4)
C9—N2—C13—C16	179.1 (3)	C6—C1—C2—C3	−177.7 (3)
C1—N1—C5—C4	−1.5 (4)	C9—C10—C11—C12	0.5 (4)
C1—N1—C5—C8	178.6 (3)	C9—C10—C11—C15	−178.2 (3)
C5—N1—C1—C2	0.5 (4)	C13—C12—C11—C10	−0.7 (4)
C5—N1—C1—C6	179.1 (2)	C13—C12—C11—C15	178.0 (3)
N2—C13—C12—C11	0.7 (4)	C1—C2—C3—C4	−0.9 (4)
C16—C13—C12—C11	−178.9 (3)	C1—C2—C3—C7	177.3 (3)
C13—N2—C9—C10	0.4 (4)	C2—C3—C4—C5	−0.1 (4)
C13—N2—C9—C14	−179.6 (3)	C7—C3—C4—C5	−178.4 (3)
N2—C9—C10—C11	−0.3 (4)	N1—C5—C4—C3	1.3 (4)
C14—C9—C10—C11	179.6 (3)	C8—C5—C4—C3	−178.8 (3)

### Hydrogen-bond geometry (Å, °)

**Table:**

*D*—H···*A*	*D*—H	H···*A*	*D*···*A*	*D*—H···*A*
O1—H1···O8	0.85 (2)	1.77 (2)	2.610 (2)	169 (3)
O3—H3···O7^i^	0.85 (3)	1.72 (3)	2.569 (2)	173 (3)
O5—H5···O4^ii^	0.84 (2)	1.79 (2)	2.627 (3)	173 (3)
O6—H6···O2	0.85 (2)	1.69 (2)	2.538 (2)	176 (3)
N1—H1A···O4^iii^	0.86	1.77	2.633 (3)	177
N2—H2A···O8^iv^	0.86	1.78	2.632 (3)	170
C2—H2B···O6^v^	0.93	2.59	3.443 (4)	152
C4—H4A···O7^i^	0.93	2.59	3.481 (4)	161

Symmetry codes: (i) *x*+1, *y*, *z*; (ii) *x*−1, *y*, *z*; (iii) −*x*+2, *y*−1/2, −*z*+1; (iv) −*x*+1, *y*+1/2, −*z*+1; (v) −*x*+1, *y*−1/2, −*z*.
